# Paternal genetic effects of cadmium exposure during pregnancy on hormone synthesis disorders in ovarian granulosa cells of offspring

**DOI:** 10.1186/s13048-023-01175-5

**Published:** 2023-05-16

**Authors:** Yi Sun, Zhangpin Liu, Wenchang Zhang, Hao Lin, Qingyu Li, Chenchen Liu, Chenyun Zhang

**Affiliations:** 1 Department of Preventive Medicine, Fujian Provincial Key Laboratory of Environmental Factors and Cancer, Key Laboratory of Environment and Health, School of Public Health, Fujian Medical University, Fuzhou, 350122 Fujian Province China; 2 Key Laboratory of Environment and Female Reproductive Health, The Eighth Affiliated Hospital, Sun Yat-sen University, Shenzhen, China; 3 Fuzhou Center for Disease Control and Prevention, Fuzhou, 350005 Fujian Province China; 4 School of Health Management, Fujian Medical University, Fuzhou, 350122 Fujian Province China

**Keywords:** Cadmium, Paternal genetic effect, Estradiol, Progesterone, miRNAs, DNA methylation

## Abstract

The aim of this study was to investigate the paternal genetic intergenerational and transgenerational genetic effects of cadmium (Cd) exposure during pregnancy on estradiol (E_2_) and progesterone (Pg) synthesis in the ovarian granulosa cells (GCs) of offspring. Pregnant SD rats were intragastrically exposed to CdCl_2_ (0, 0.5, 2.0, 8.0 mg/kg) from days 1 to 20 to produce the F1 generation, F1 males were mated with newly purchased females to produce the F2 generation, and the F3 generation was obtained in the same way. Using this model, Cd-induced hormone synthesis disorders in GCs of F1 have been observed [8]. In this study, altered serum E_2_ and Pg levels in both F2 and F3 generations showed a nonmonotonic dose‒response relationship. In addition, hormone synthesis-related genes (Star, Cyp11a1, Cyp17a1, Cyp19a1, Sf-1) and miRNAs were observed to be altered in both F2 and F3. No differential changes in DNA methylation modifications of hormone synthesis-related genes were observed, and only the Adcy7 was hypomethylated. In summary, paternal genetic intergenerational and transgenerational effects exist in ovarian GCs E_2_ and Pg synthesis disorders induced by Cd during pregnancy. In F2, the upregulation of StAR and CYP11A1, and changes in the miR-27a-3p, miR-27b-3p, and miR-146 families may be important, while changes in the miR-10b-5p and miR-146 families in F3 may be important.

**Supplementary Information:**

The online version contains supplementary material available at 10.1186/s13048-023-01175-5.

## Introduction

Cadmium (Cd) is a heavy metal that is widely used in industry, especially in the electronics, chemical and nuclear industries, that easily enters the environment through waste gas, wastewater, waste residue and other forms to cause pollution [1, 2]. Cd in the environment is not biodegradable and can enter the human body through the food chain and accumulate in the body after adsorption by crops, resulting in irreversible damage to multiple organ systems of the human body; the reproductive toxicity damage of Cd has been very clear [3], and significant ovarian toxicity effects can occur during different periods of Cd exposure [4–6]. In addition, Cd can also cross the placental barrier to effect offspring [7]. Our group previously showed that Cd exposure during pregnancy can induce estradiol (E_2_) and progesterone (Pg) synthesis disorders in ovarian granulosa cells (GCs) of F1 female rats, and there are significant maternal genetic intergenerational and transgenerational effects [8]. Cd exposure during pregnancy can also cause significant reproductive damage in F1 and F2 male rats [9]. Emerging issues of concern in this study are as follows: It is well-established that male offspring are affected following Cd exposure during pregnancy, but are ovarian GCs are compromised in their female offspring? That is, do intergenerational and transgenerational effects of paternal inheritance present as offspring ovarian GCs hormone synthesis disorders caused by Cd exposure during pregnancy.

Ovarian GCs are important functional cell populations for steroid hormone (Pg, E_2_) synthesis in the ovary. Star, Cyp11a1, Cyp17a1, and Cyp19a1 are key genes in E_2_ and Pg synthesis, and Sf-1 stimulates the expression of almost all genes involved in cholesterol mobilization and steroid hormone biosynthesis [10]. Many studies have shown that the expression of hormone synthesis genes changes to different extents when steroid hormone levels change after exposure to chemicals in the body, so genes in the hormone synthesis pathway are often important targets for chemicals, which affects hormone synthesis [11, 12]. During hormone synthesis disorder in ovarian GCs of F1 offspring exposed to Cd during pregnancy, the above hormone synthesis-related genes also showed different degrees of changes and were closely related to hormone phenotypic changes [8]. Therefore, this study will also focus on the expression of hormone synthesis-related genes during paternal inheritance of hormone synthesis disorders in ovarian GCs induced by Cd exposure during pregnancy.

Epigenetic modifications include but are not limited to DNA methylation, histone modifications, noncoding RNAs (ncRNAs), and chromatin structure alterations capable of regulating gene expression in multiple critical physiological and pathological processes [13]. MicroRNAs (miRNAs) play an important role in the regulation of E_2_ and Pg synthesis in ovarian GCs. MiR-132 has been found to target and suppress the mRNA expression of Star which in turn suppresses steroid hormone production [14]; miR-31, which targets HSD17B14 and FSHR, affects apoptosis and steroid hormone metabolism in porcine ovarian GCs [15]; and miR-130a-3p regulates steroid hormone synthesis in goat ovarian GCs by targeting the PMEPA1 gene [16]. In addition, DNA methylation modifications are also important for the expression of genes involved in E_2_ and Pg synthesis. Expression of Cyp17a1 has been found to be regulated by methylation of CpG islands in its promoter region [17]; during follicular cell growth and development, follicle formation and luteinization, methylation patterns of Cyp19a1 are essential to maintain its normal function [18]. Previous studies have revealed the maternal genetic intergenerational and transgenerational effects of Cd exposure during pregnancy on apoptosis in ovarian GCs of offspring, which is accompanied by significant changes in miRNAs and DNA methylation profiles [19]. Therefore, this study also focused on changes in miRNAs and DNA methylation in offspring ovarian GCs following paternal genetic effect of Cd exposure during pregnancy on hormone synthesis.

In summary, the aim of this study was to observe whether this impairment of hormone synthesis in F1 female offspring induced by Cd exposure during pregnancy can be transmitted paternally to female offspring and whether there are intergenerational and transgenerational effects of paternal inheritance. What are the changes in hormone synthesis-related genes, miRNAs, and DNA methylation modifications in ovarian GCs resulting from this genetic effect? This study can provide an important scientific basis for further study of Cd-induced ovarian toxicity multigenerational genetic effects, and epigenetic regulation mechanisms.

## Methods

### Animal model establishment

SPF adult Sprague‒Dawley rats (64 females, 32 males) were purchased from Shanghai SLAC Laboratory Animal Co., Ltd. (licence No. SCXK, 2012-0002). After one week of adaptive feeding, the rats were cohabited at a ratio of 2:1, and vaginal plugs or vaginal smears were examined the next day. If a vaginal plug or sperm was found, the rats were regarded as pregnant, and the pregnant female rats were fed separately. Pregnant rats were randomly divided into 4 groups according to body weight, and exposed to 0, 0.5, 2.0, and 8.0 mg/kg CdCl_2_ every day for the whole course of pregnancy (GD1-GD20) by equal volume gavage (1 ml/300 g), and the F1 generation was delivered spontaneously. The F1 generation was routinely housed until adulthood (56 days old), and one male rat from each litter was randomly selected and cohabited with a newly purchased healthy female rat to produce F2 offspring at a male to female ratio of 1:1. The F3 generation was obtained in the same way as the F2 generation. After F2 and F3 female rats were routinely maintained until adulthood (56 days old), blood and ovarian GCs were collected during estrus. All rats were housed in a barrier environment (temperature 22 ± 1 °C, relative humidity 50 ± 5%).

### Cultivation of rat ovarian GCs

Adult female Sprague‒Dawley rats were sacrificed during estrus. After alcohol disinfection of the back fur, bilateral ovaries were rapidly removed from the back of the rats under aseptic conditions, washed in DMEM/F12 culture medium containing double antibodies (100 U/ml penicillin, 100 mg/L streptomycin), and incubated at 37 °C to remove fat and blood; then, new prewarmed DMEM/F12 culture medium was replaced. Follicles were punctured with a 25-gauge needle under a stereomicroscope to direct granulosa outflow. All the culture medium was collected and allowed to stand for 5 min. The supernatant was transferred to another centrifuge tube centrifuged at 1000 rpm for 5 min, and the supernatant was discarded. The cell pellet was made into suspension with DMEM/F12, and the cell suspension was slowly added to the prepared 50% Percoll separation solution at a ratio of 1:1 and centrifuged at 400 g for 20 min. The middle white granular cell layer was aspirated and washed with PBS to obtain a pure granular cell pellet. A single cell suspension was prepared by adding an appropriate amount of DMEM/F12 + 10% FBS medium containing double antibodies to the pellet, and the cell concentration was adjusted to 1 × 10^6^ cells/mL; samples were then transferred to a culture dish and placed in a 37 °C cell incubator for 48 h [20, 21].

### ELISA measurement serum E_2_ and pg levels

Serum was collected from F2 and F3 females (n = 8) to measure hormone levels (E2 and Pg). Enzyme-linked immunosorbent assay (ELISA) kits (Elabscience Biotechnology, Wuhan, China) were used to measure the concentrations of these hormones at 450 nm. Details are provided in previous studies [22].

### Reverse transcription and real-time quantitative PCR

Total RNA from F2 and F3 ovarian GCs (n = 6/per group) was extracted by using TRIzol reagent (Invitrogen, California, USA). Two micrograms of RNA was reverse transcribed into cDNA by the PrimeScript™ RT Kit or the Mir-XmiRNA First Strand Synthesis Kit (Takara Biotechnology, Dalian, China). qRT‒PCR was performed on a Light Cycler 480 real-time PCR system (Roche, Switzerland) with RR420 (for mRNA) or RR820 (for miRNA) (Takara Bio Inc., Shiga, Japan). β-actin or U6 was used as an internal control. Table S1 lists the primers used for qRT‒PCR.

### Western blotting analysis

Total protein was extracted from F2 and F3 generation rat GCs (n = 6/group), and the total protein concentration was determined by a BCA kit (Beyotime Institute of Biotechnology, China). SDS‒PAGE gels were prepared, and protein samples were loaded, processed by electrophoresis and transferred to a PVDF membrane. Following incubation with primary and secondary antibodies, the membranes were visualized by enhanced chemiluminescence [19].

### DNA methylation sequencing

Ovarian GCs (n = 3/per group) from the F2 control and 8.0 mg/kg groups were used for DNA methylation sequencing. Samples were digested by the methylation-insensitive restriction enzyme, Mspl. The digested DNA fragments were end-repaired, A-tailed, and ligated with sequencing adapters; DNA fragments with inserts ranging in length from 150 to 300 bp were selected by gel cutting; followed by bisulfite treatment (EZ DNA Methylation Gold Kit, Zymo Research) and PCR amplification to obtain DNA libraries. After quality inspection, Illumina HiSeq was performed. Four fluorescently labelled dNTPs, DNA polymerase and adaptor primers were added to the sequenced flow cell for amplification. When each sequencing cluster extended the complementary strand, each fluorescently labelled dNTP was added and emitted its corresponding fluorescence. The sequencer captured the fluorescence signal and converted the light signal into the sequencing peak through computer software to obtain the sequence information of the fragment to be tested.

### Statistical analysis

Statistical analysis was performed by SPSS 21.0, quantitative data were compared by t-test or one-way analysis of variance, and the LSD post-hoc test was used for comparisons between groups. Graphs were generated using GraphPad Prism 8.0. *P* < 0.05 was considered statistically significant.

## Results

### Serum E_2_ and pg levels during estrus in F2 and F3 adult female rats

**Fig. 1:**
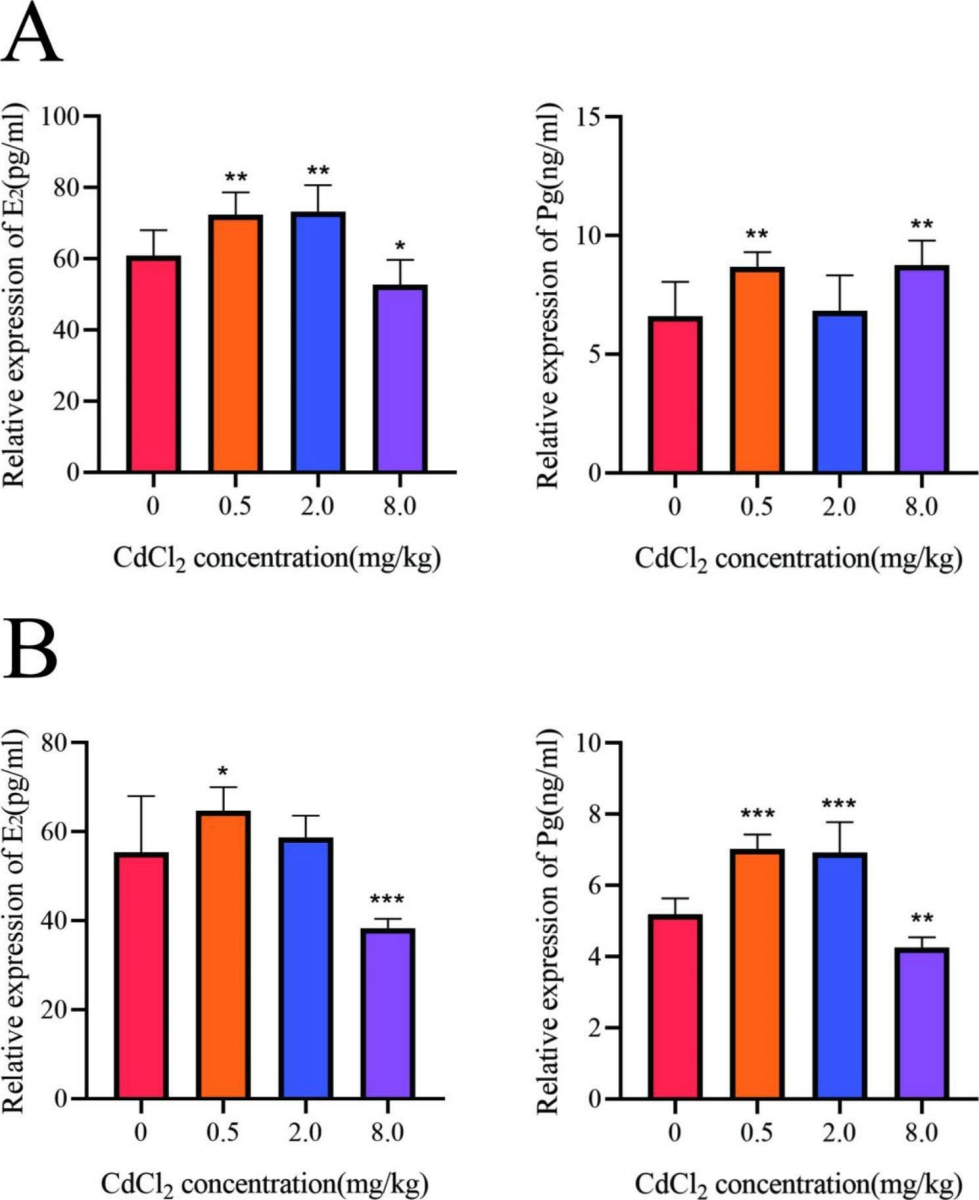
Serum E_2_ and Pg levels during estrus in F2 and F3 adult females, *, *P* < 0.05, **, *P* < 0.01, ***, *P* < 0.001, n = 8. (A: F2 generation; B: F3 generation)

Serum E_2_ and Pg levels were measured by ELISA in F2 and F3 adult female rats during estrus. In the F2 generation, compared with the control group, E_2_ levels increased in the 0.5 mg/kg and 2.0 mg/kg groups and decreased in the 8.0 mg/kg group (*P* < 0.05), and serum Pg levels increased in the 0.5 mg/kg and 8.0 mg/kg groups (*P* < 0.05) (Fig. 1A). In the F3 generation, compared with the control group, serum E_2_ levels increased in the 0.5 mg/kg group and decreased in the 8.0 mg/kg group (*P* < 0.05); serum Pg levels increased in the 0.5 mg/kg and 2.0 mg/kg groups and decreased in the 8.0 mg/kg group (*P* < 0.05) (Fig. 1B).

### Expression of E_2_ and pg synthesis-related genes in F2 and F3 ovarian GCs

**Fig. 2:**
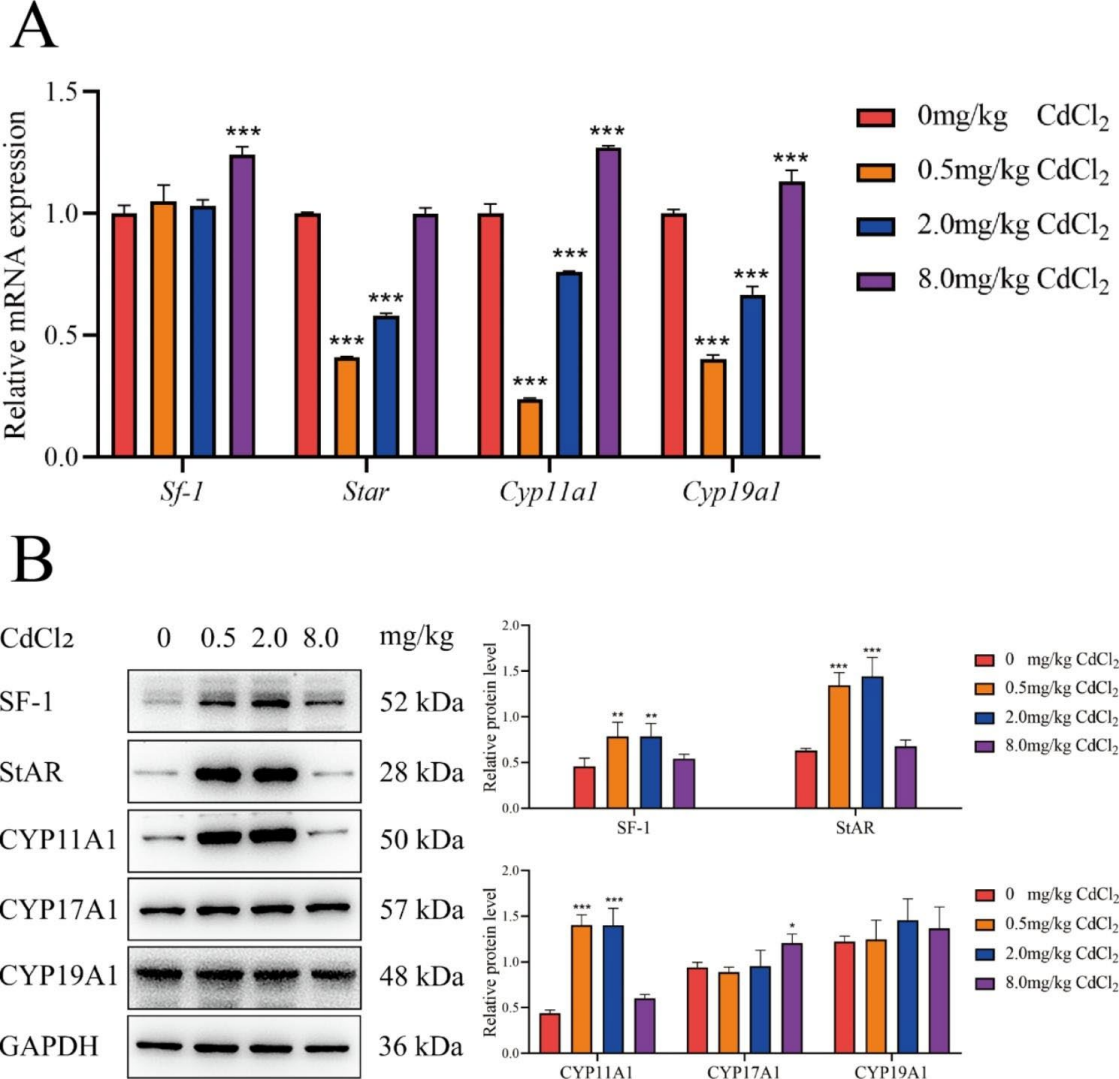
mRNA and protein expression levels of E_2_ and Pg synthesis-related genes in ovarian GCs of F2 adult female rats, *, *P* < 0.05, **, *P* < 0.01, ***, *P* < 0.001, n = 6. (A: mRNA expression level; B: protein expression level)

The mRNA and protein expression levels of hormone synthesis-related genes (Sf-1, Star, Cyp11a1, Cyp17a1, Cyp19a1) in F2 and F3 rat ovarian GCs were measured by qRT‒PCR and western-blotting. In the F2 generation, compared with the control group, the mRNA expression levels of Cyp11a1 and Cyp19a1 were decreased in the 0.5 mg/kg and 2.0 mg/kg groups but increased in the 8.0 mg/kg group; the mRNA expression levels of Sf1 were increased in the 8.0 mg/kg group, and that of Star was decreased in the 0.5 mg/kg and 2.0 mg/kg groups (all *P* < 0.05). Compared with the control group, the protein expression levels of SF-1, StAR, and CYP11A1 were increased in the 0.5 mg/kg and 2.0 mg/kg groups; the protein expression levels of CYP17A1 were increased in the 8.0 mg/kg group (all *P* < 0.05) (Fig. 2).

**Fig. 3:**
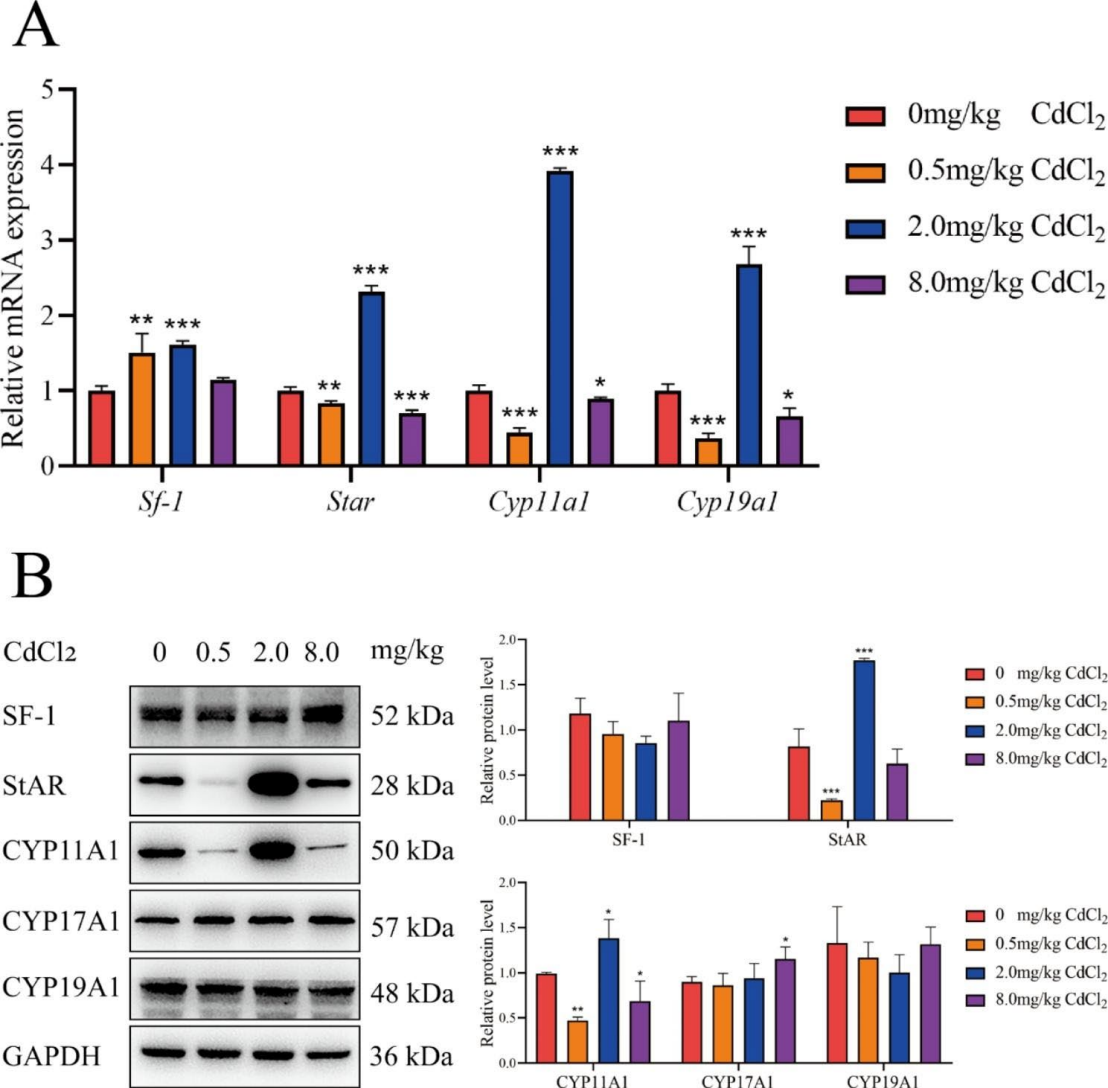
mRNA and protein expression levels of E_2_ and Pg synthesis-related genes in ovarian GCs of F3 adult female rats, *, *P* < 0.05, **, *P* < 0.01, ***, *P* < 0.001, n = 6. (A: mRNA expression level; B: protein expression level)

In the F3 generation, compared with the control group, the mRNA expression of Star, Cyp11a1, and Cyp19a1 was increased in the 2.0 mg/kg group but decreased in the 0.5 mg/kg and 8.0 mg/kg groups; the mRNA expression levels of sf1 were increased in the 0.5 mg/kg and 2.0 mg/kg groups (all *P* < 0.05). Compared with the control group, the protein expression levels of StAR were decreased in the 0.5 mg/kg group and increased in the 8.0 mg/kg group; the protein expression levels of CYP11A1 were decreased in the 0.5 mg/kg and 8.0 mg/kg groups and increased in the 2.0 mg/kg group; and the protein expression levels of CYP17A1 were increased in the 8.0 mg/kg group (all *P* < 0.05) (Fig. 3).

### Screening and validation of E_2_ and pg synthesis-related miRNAs in F2 and F3 ovarian GCs

Based on the previous miRNA microarray data, database prediction, relevant literature reports and previous findings, a total of 24 E_2_ and Pg synthesis-related miRNAs (as shown in Table 1) were selected and measured by qRT‒PCR.

**Table 1:** Screening results of miRNAs

miRNAs	Comprehensive screening results
rno-miR-10b-5p	*Star*, TargetScan, miRDB, miRWalk, preliminary study results
rno-miR-125b-1-3p	*Sf-1*, *Star*, *Cyp11a1*, *Cyp19a1*, previous miRNA microarray, miRWalk
rno-miR-138-5p	*Sf-1*, TargetScan, RNAhybrid
rno-miR-146a-5p	*Star*, miRDB, RNAhybrid
rno-miR-146b-5p	*Star*, TargetScan, miRDB, RNAhybrid *Cyp19a1*, literature Reports
rno-miR-146a-3p	*Star*, TargetScan, RNAhybrid
rno-miR-152-3p	*Star*, TargetScan, RNAhybrid
rno-miR-1839-5p	*Star*, TargetScan, RNAhybrid
rno-miR-1896	*Cyp17a1*, miRWalk, RNAhybrid
rno-miR-207	*Star*, *Cyp19a1*, TargetScan, RNAhybrid, miRWalk
rno-miR-210-3p	*Sf-1*, *Cyp11a1*, *Cyp17a1*, *Cyp19a1*, miRWalk, previous miRNA microarray
rno-miR-211-3p	*Sf-1*, TargetScan, miRDB, RNAhybrid
rno-miR-212-5p	*Sf-1*, *Star*, TargetScan, RNAhybrid
rno-miR-24-3p	*Cyp11a1*, *Cyp17a1*, miRWalk, RNAhybrid
rno-miR-27a-3p	*Sf-1*, *Star*, *Cyp11a1*, TargetScan, RNAhybrid, preliminary study results
rno-miR-27b-3p	*Sf-1*, *Star*, *Cyp11a1*, TargetScan, RNAhybrid
rno-miR-27a-5p	*Sf-1*, *Star*, *Cyp11a1, Cyp19a1*, TargetScan, RNAhybrid, miRWalk
rno-miR-3558-5p	*Star*, TargetScan, RNAhybrid, previous miRNA microarray
rno-miR-486	*Star*, TargetScan, RNAhybrid
rno-miR-673-5p	*Sf-1*, *Star*, *Cyp11a1*, TargetScan, RNAhybrid
rno-miR-702-3p	*Sf-1*, TargetScan, miRDB, RNAhybrid
rno-miR-185-5p	*Sf-1*, *Star*, TargetScan, RNAhybrid, previous miRNA microarray
rno-miR-32-3p	*Sf-1*, *Star*, TargetScan, RNAhybrid, previous miRNA microarray
rno-miR-465-5p	*Sf-1*, *Star*, *Cyp19a1*, TargetScan、RNAhybrid, previous miRNA microarray

In the F2 generation, compared with the control group, the expression of 138-5p, miR-1839-5p, miR-32-3p and miR-27b-3p was increased in all dose groups; the expression of miR-210-3p and miR-27a-3p was increased in the 2.0 mg/kg and 8.0 mg/kg groups; the expression of miR-152-3p was increased in the 0.5 mg/kg and 8.0 mg/kg groups; the expression of miR-125b-1-3p, miR-24-3p and miR-27a-5p was increased in the 8.0 mg/kg group; the expression of miR-146a-3p and miR-146a-5p was increased in the 0.5 mg/kg and 2.0 mg/kg groups; and the expression of miR-146b-5p was increased only in the 0.5 mg/kg group (all *P* < 0.05). Compared with the control group, the expression of miR-1896, miR-207 and miR-211-3p was decreased in all dose groups; the expression of miR-10b-5p, miR-212-5p, and miR-673-5p was decreased in the 0.5 mg/kg and 2.0 mg/kg groups; miR-702-3p was decreased in the 2.0 mg/kg group; miR-3558-5p was decreased in the 0.5 mg/kg group; miR-185-5p was decreased in the 0.5 mg/kg group but increased in the 8.0 mg/kg group; and miR-486 expression was increased in the 0.5 mg/kg group but decreased in the 2.0 mg/kg and 8.0 mg/kg groups (all *P* < 0.05) (Table 2).

**Table 2:** Expression of estrogen and progesterone synthesis-related miRNAs in F2 ovarian GCs

Group (mg/kg)	0	0.5	2.0	8.0
rno-miR-10b-5p	1.010±0.172	0.625±0.125^**^	0.575±0.019^**^	1.159±0.133
$\overline {\text{X}}$± 1.96 S	0.673–1.347	0.380–0.870	0.538–0.612	0.898–1.420
rno-miR-125b-1-3p	1.000±0.049	0.874±0.084	1.117±0.021	1.175±0.098^*^
$\overline {\text{X}}$± 1.96 S	0.904–1.096	0.709–1.039	0.952–1.158	0.983–1.367
rno-miR-138-5p	1.006±0.128	2.284±0.190^***^	2.541±0.395^***^	3.512±0.278^***^
$\overline {\text{X}}$± 1.96 S	0.755–1.257	1.912–2.656	1.767–3.315	2.967–4.057
rno-miR-146a-5p	1.001±0.056	4.122±0.207^***^	2.078±0.220^***^	0.880±0.045
$\overline {\text{X}}$± 1.96 S	0.891–1.111	3.716–4.528	1.647–2.509	0.792–0.968
rno-miR-146b-5p	1.000±0.002	1.564±0.203^***^	1.070±0.094	0.944±0.047
$\overline {\text{X}}$± 1.96 S	0.996–1.004	1.166–1.962	0.886–1.254	0.852–1.036
rno-miR-146a-3p	1.013±0.203	3.076±0.253^***^	2.423±0.208^***^	1.014±0.147
$\overline {\text{X}}$± 1.96 S	0.615–1.411	2.580–3.572	2.015–2.831	0.726–1.302
rno-miR-152-3p	1.000±0.027	1.436±0.285^*^	1.309±0.184	3.453±0.085^***^
$\overline {\text{X}}$± 1.96 S	0.947–1.053	0.877–1.99	0.948–1.670	3.286–3.620
rno-miR-1839-5p	1.001±0.059	1.584±0.159^***^	1.260±0.117^*^	1.643±0.077^***^
$\overline {\text{X}}$± 1.96 S	0.885–1.117	1.272–1.896	1.031–1.489	1.492–1.794
rno-miR-1896	1.006±0.129	0.717±0.095^**^	0.469±0.032^***^	0.623±0.080^**^
$\overline {\text{X}}$± 1.96 S	0.753–1.259	0.531–0.903	0.406–0.532	0.467–0.780
rno-miR-207	1.000±0.038	0.793±0.085^**^	0.687±0.028^***^	0.799±0.084^**^
$\overline {\text{X}}$± 1.96 S	0.926–1.074	0.626–0.960	0.632–0.742	0.634–0.964
rno-miR-210-3p	1.000±0.027	1.083±0.085	1.251±0.080^**^	1.718±0.099^***^
$\overline {\text{X}}$± 1.96 S	0.947–1.053	0.916–1.250	1.094–1.408	1.524–1.912
rno-miR-211-3p	1.011±0.178	0.611±0.018^**^	0.663±0.036^**^	0.589±0.049^**^
$\overline {\text{X}}$± 1.96 S	0.662–1.360	0.576–0.646	0.591–0.733	0.493–0.685
rno-miR-212-5p	1.005±0.125	0.852±0.047^*^	0.823±0.048^*^	0.983±0.025
$\overline {\text{X}}$± 1.96 S	0.760–1.250	0.760–0.944	0.729–0.917	0.934–1.032
rno-miR-24-3p	1.024±0.255	1.192±0.209	1.096±0.189	2.554±0.254^***^
$\overline {\text{X}}$± 1.96 S	0.524–1.524	0.782–1.602	0.726–1.466	2.056–3.052
rno-miR-27a-3p	1.003±0.090	0.880±0.143	1.512±0.081^**^	1.806±0.254^***^
$\overline {\text{X}}$± 1.96 S	0.827–1.179	0.600–1.160	1.353–1.671	1.308–2.304
rno-miR-27b-3p	1.001±0.055	1.298±0.084^**^	1.260±0.086^**^	1.609±0.086^***^
$\overline {\text{X}}$± 1.96 S	0.893–1.109	1.133–1.463	1.091–1.429	1.440–1.778
rno-miR-27a-5p	1.020±0.253	0.928±0.149	0.935±0.086	1.697±0.339^**^
$\overline {\text{X}}$± 1.96 S	0.524–1.516	0.636–1.220	0.766–1.104	1.033–2.361
rno-miR-3558-5p	1.026±0.269	0.539±0.076^*^	0.840±0.294	1.384±0.131
$\overline {\text{X}}$± 1.96 S	0.499–1.553	0.390–0.688	0.264–1.416	1.127–1.641
rno-miR-673-5p	1.009±0.168	0.716±0.069^*^	0.596±0.158^**^	0.988±0.060
$\overline {\text{X}}$± 1.96 S	0.680–1.338	0.581–0.851	0.286–0.906	0.870–1.106
rno-miR-702-3p	1.025±0.291	0.827±0.156	0.536±0.031^*^	1.103±0.186
$\overline {\text{X}}$± 1.96 S	0.455–1.595	0.521–1.133	0.475–0.597	0.738–1.468
rno-miR-185-5p	1.009±0.164	0.636±0.011^*^	1.214±0.336	2.723±0.096^***^
$\overline {\text{X}}$± 1.96 S	0.688–1.330	0.614–0.658	0.555–1.873	2.535–2.911
rno-miR-32-3p	1.001±0.066	2.525±0.161^***^	1.510±0.200^**^	2.572±0.098^***^
$\overline {\text{X}}$± 1.96 S	0.872–1.130	2.209–2.841	1.118–1.902	2.380–2.764
rno-miR-465-5p	1.008±0.155	1.032±0.041	0.883±0.258	0.956±0.123
$\overline {\text{X}}$± 1.96 S	0.704–1.312	0.952–1.112	0.377–1.389	0.715–1.197
rno-miR-486	1.001±0.045	1.245±0.037^**^	0.832±0.012^*^	0.746±0.118^**^
$\overline {\text{X}}$± 1.96 S	0.913–1.089	1.172–1.318	0.808–0.856	0.515–0.977

Note: Compared with control group, *, *P* < 0.05, **, *P* < 0.01, ***, *P* < 0.001, n = 6

In the F3 generation, compared with the control group, the expression of miR-152-3p and miR-32-3p was increased in each dose group; the expression of miR-10b-5p and miR-702-3p was increased in the 0.5 mg/kg and 8.0 mg/kg groups; the expression of miR-212-5p was increased in the 8.0 mg/kg group; the expression of miR-210-3p and miR-185-5p was increased in the 2.0 mg/kg group; and the expression of miR-1839-5p was increased in the 0.5 mg/kg group and decreased in the 8.0 mg/kg group (all *P* < 0.05). Compared with the control group, the expression of miR-207 was decreased in each dose group; the expression of miR-146a-5p and miR-146b-5p was decreased in the 2.0 mg/kg and 8.0 mg/kg groups; the expression of miR-1896 was decreased in the 0.5 mg/kg and 2.0 mg/kg groups; and the expression of miR-27a-3p and miR-27b-3p was decreased in the 2.0 mg/kg group; miR-24-3p and miR-27a-5p expression was decreased in the 0.5 mg/kg group; miR-125b-1-3p expression was decreased in the 0.5 mg/kg group but increased in the 2.0 mg/kg and 8.0 mg/kg groups; miR-138-5p expression was decreased in the 2.0 mg/kg group but increased in the 0.5 mg/kg and 8.0 mg/kg groups; miR-211-3p expression was decreased in the 2.0 mg/kg group but increased in the 8.0 mg/kg group; and miR-3558-5p and miR-486 expression was decreased in the 0.5 mg/kg group but increased in the 2.0 mg/kg and 8.0 mg/kg groups (all *P* < 0.05) (Table 3).

**Table 3:** Expression of estrogen and progesterone synthesis-related miRNAs in F3 ovarian GCs

Group (mg/kg)	0	0.5	2.0	8.0
rno-miR-10b-5p	1.003±0.100	1.572±0.167^**^	0.806±0.124	1.352±0.132^*^
$\overline {\text{X}}$± 1.96 S	0.807–1.199	1.245–1.899	0.563–1.049	1.093–1.611
rno-miR-125b-1-3p	1.000±0.034	0.873±0.0120^*^	1.302±0.076^***^	1.122±0.059^*^
$\overline {\text{X}}$± 1.96 S	0.933–1.067	0.638–1.108	1.153–1.451	1.006–1.238
rno-miR-138-5p	1.000±0.014	1.164±0.057^**^	0.745±0.091^***^	1.333±0.011^***^
$\overline {\text{X}}$± 1.96 S	0.973–1.027	1.052–1.276	0.567–0.923	1.311–1.355
rno-miR-146a-5p	1.000±0.011	0.875±0.153	0.390±0.050^***^	0.560±0.110^**^
$\overline {\text{X}}$± 1.96 S	0.978–1.022	0.575–1.175	0.292–0.488	0.344–0.776
rno-miR-146b-5p	1.003±0.097	1.019±0.209	0.519±0.063^**^	0.663±0.003^**^
$\overline {\text{X}}$± 1.96 S	0.813–1.193	0.609–1.429	0.396–0.642	0.657–0.669
rno-miR-146a-3p	1.000±0.052	1.094±0.099	0.382±0.038^***^	0.538±0.114^***^
$\overline {\text{X}}$± 1.96 S	0.898–1.102	0.900-1.288	0.308–0.456	0.315–0.761
rno-miR-152-3p	1.002±0.068	1.226±0.108^**^	1.165±0.072^*^	1.235±0.058^**^
$\overline {\text{X}}$± 1.96 S	0.869–1.135	1.014–1.438	1.024–1.306	1.121–1.349
rno-miR-1839-5p	1.000±0.033	1.731±0.165^***^	0.815±0.065	0.761±0.110^*^
$\overline {\text{X}}$± 1.96 S	0.935–1.065	1.408–2.054	0.688–0.942	0.545–0.977
rno-miR-1896	1.003±0.098	0.538±0.027^***^	0.637±0.067^***^	0.894±0.016
$\overline {\text{X}}$± 1.96 S	0.811–1.195	0.485–0.59	0.506–0.768	0.863–0.925
rno-miR-207	1.003±0.095	0.833±0.069^*^	0.688±0.028^***^	0.660±0.049^***^
$\overline {\text{X}}$± 1.96 S	0.817–1.189	0.698–0.968	0.633–0.743	0.564–0.756
rno-miR-210-3p	1.002±0.068	0.836±0.050	1.222±0.112^*^	0.826±0.129
$\overline {\text{X}}$± 1.96 S	0.869–1.135	0.738–0.934	1.002–1.442	0.573–1.079
rno-miR-211-3p	1.008±0.150	0.934±0.154	0.601±0.002^**^	1.231±0.015^*^
$\overline {\text{X}}$± 1.96 S	0.714–1.302	0.632–1.236	0.597–0.605	1.202–1.260
rno-miR-212-5p	1.002±0.069	0.879±0.110	0.927±0.075	1.148±0.020^*^
$\overline {\text{X}}$± 1.96 S	0.867–1.137	0.663–1.095	0.780–1.074	1.109–1.187
rno-miR-24-3p	1.001±0.065	0.623±0.140^**^	1.027±0.147	0.779±0.163
$\overline {\text{X}}$± 1.96 S	0.874–1.128	0.349–0.897	0.739–1.315	0.460–1.098
rno-miR-27a-3p	1.000±0.023	0.879±0.112	1.201±0.123^*^	1.056±0.059
$\overline {\text{X}}$± 1.96 S	0.955–1.045	0.659–1.099	0.960–1.442	0.940–1.172
rno-miR-27b-3p	1.002±0.085	0.914±0.047	1.218±0.007^**^	0.926±0.007
$\overline {\text{X}}$± 1.96 S	0.835–1.169	0.822–1.006	1.204–1.232	0.912–0.940
rno-miR-27a-5p	1.018±0.227	0.710±0.112^*^	1.155±0.074	0.765±0.093
$\overline {\text{X}}$± 1.96 S	0.573–1.463	0.490–0.930	1.010–1.300	0.583–0.947
rno-miR-3558-5p	1.000±0.020	0.561±0.057^**^	1.556±0.188^**^	1.578±0.237^**^
$\overline {\text{X}}$± 1.96 S	0.961–1.039	0.449–0.673	1.188–1.924	1.113–2.043
rno-miR-673-5p	1.018±0.224	0.766±0.150	1.120±0.129	0.954±0.177
$\overline {\text{X}}$± 1.96 S	0.579–1.457	0.472–1.060	0.867–1.373	0.607–1.301
rno-miR-702-3p	1.004±0.111	1.947±0.288^***^	1.292±0.059	1.591±0.227^**^
$\overline {\text{X}}$± 1.96 S	0.786–1.222	1.383–2.511	1.176–1.408	1.146–2.036
rno-miR-185-5p	1.006±0.135	1.091±0.151	2.055±0.137^***^	1.004±0.162
$\overline {\text{X}}$± 1.96 S	0.741–1.271	0.795–1.387	1.786–2.324	0.686–1.322
rno-miR-32-3p	1.001±0.057	0.647±0.088^*^	1.467±0.289^**^	1.302±0.004^*^
$\overline {\text{X}}$± 1.96 S	0.889–1.113	0.475–0.819	0.901–2.033	1.294–1.310
rno-miR-465-5p	1.000±0.025	0.550±0.104^**^	1.221±0.135^*^	1.246±0.156^*^
$\overline {\text{X}}$± 1.96 S	0.951–1.049	0.346–0.754	0.956–1.486	0.940–1.552
rno-miR-486	1.001±0.065	0.499±0.003^**^	1.429±0.213^**^	1.102±0.009
$\overline {\text{X}}$± 1.96 S	0.874–1.128	0.493–0.505	1.012–1.846	1.084–1.120

Note: Compared with control group, *, *P* < 0.05, **, *P* < 0.01, ***, *P* < 0.001, n = 6

## Methylation pattern of genome-wide DNA promoter in ovarian GCs

The results of genome-wide DNA methylation sequencing in GCs of the F2 generation showed that the mean CpG methylation level was 55.64% in the control group and 56.82% in the 8.0 mg/kg group. Differentially methylated region (DMR) analysis with Metilence software revealed a total of 593 DMRs, of which, the expression of 303 DMRs were upregulated and 290 were downregulated. Regional annotation of DMRs revealed a total of 86 differentially methylated gene promoter regions, 39 hypermethylated and 47 hypomethylated. However, Sf-1, Star, Cyp11a1, Cyp11a1, and Cyp19a1 were not found to be differentially methylated in their promoter regions.

Among genes that were differentially methylated, Adcy7, which was hypomethylated, is associated with E_2_ and Pg synthesis in ovarian GCs. Adcy7 belongs to a class of adenylyl cyclase’s that can regulate the expression of Star and Cyp11a1 through the cAMP/PKA signalling pathway. Compared with the control group, the mRNA expression level of Adcy7 was increased in the 0.5 mg/kg and 8.0 mg/kg groups in the F2 generation and increased in each dose group in the F3 generation (*P* < 0.05) (Table 4).

**Table 4:** mRNA expression level of Adcy7 in GCs of F2 and F3 generations

Group (mg/kg)	*Adcy7* (F2)	*Adcy7* (F3)
0	1.003±0.092	1.003±0.092
$\overline {\text{X}}$± 1.96 S	0.823–1.183	0.823–1.183
0.5	1.490±0.056^***^	1.317±0.081^**^
$\overline {\text{X}}$± 1.96 S	1.380–1.600	1.160–1.474
2.0	1.084±0.136	2.794±0.120^***^
$\overline {\text{X}}$± 1.96 S	0.817–1.351	2.559–3.029
8.0	1.875±0.066^***^	1.254±0.077^*^
$\overline {\text{X}}$± 1.96 S	1.746–2.004	1.103–1.405

Note: Compared with control group, *, *P* < 0.05, **, *P* < 0.01, ***, *P* < 0.001, n = 6

## Discussion

The health hazard of environmental Cd pollution is a major public health problem in the world today, and its prevention and control still face major challenges. In the past 20 years, we have systematically investigated the reproductive toxicity of Cd in female gonads and its related epigenetic mechanisms, but there are still few studies on ovarian toxicity damage and its genetic effects in offspring caused by Cd exposure. We have previously shown that Cd exposure during pregnancy can cause ovarian GCs damage in offspring with clear maternal genetic intergenerational and transgenerational effects [8, 19], the F1 and F2 males also showed significant reproductive impairment [9]. In this study, we investigated whether the intergenerational and transgenerational effects of abnormal E_2_ and Pg synthesis in ovarian GCs of female offspring could be induced by Cd exposure during pregnancy through paternal inheritance. The results showed that alterations in the levels of steroid hormone (E_2_ and Pg) synthesis were observed in F2 and F3 adult females. Genes and miRNAs associated with ovarian GCs hormone synthesis were also markedly altered during this genetic effect. Several relevant issues are discussed as follows:.

### Cd exposure during pregnancy can cause abnormal ovarian GCs hormone synthesis in offspring through paternal inheritance

We have previously shown that Cd exposure during pregnancy can cause toxic effects in the gonads of offspring males, and this study further observed the hormone synthesis function of ovarian GCs in F2 female offspring after F1 male gonadal injury induced by Cd exposure during pregnancy. The results showed that compared with the control group, although serum E_2_ was increased in the 0.5 mg/kg and 2.0 mg/kg groups, it was significantly decreased in the 8.0 mg/kg group; while Pg was significantly increased in the 0.5 mg/kg and 8.0 mg/kg groups, but not in the 2 mg/kg group. These results suggest that there is a paternal genetic intergenerational effect on hormone synthesis disorder in ovarian GCs of offspring induced by Cd exposure during pregnancy. We noticed a non-monotonic dose-response relationship between changes in serum hormone levels and exposure dose. This phenomenon has also been reported in some studies by others [23]. It has been shown that DEHP exposure during pregnancy showed a nonmonotonic dose‒response relationship between the changes in serum E_2_ levels in F1 females, serum Pg levels in F2 females and the number of follicles at different stages of the F3 generation and the exposure dose [24]. Nasiadek et al. found that the thickness of the endometrium did not gradually increase or decrease with increasing Cd exposure doses in female rats exposed to Cd. At a low dose (0.09 mg/kg), endometrial thickening was observed, and at a higher dose (4.5 mg/kg), endometrial atrophy was evident [25]. At present, the specific mechanism of the nonmonotonic dose response has not been fully clarified.

In this study, we further observed the changes of hormone synthesis levels in ovarian GCs of F3 female offspring produced by mating F2 male rats with normal female rats. The results showed that E_2_ was increased in 0.5 mg/kg group and decreased in 8.0 mg/kg group, Pg was increased in 0.5 mg/kg and 2.0 mg/kg groups, and decreased in 8.0 mg/kg group compared with control group. These results suggest that male injury induced by Cd exposure during pregnancy can lead to transgenerational effects in abnormal ovarian GCs hormone synthesis in offspring through paternal inheritance. The pattern of hormonal changes was also non-monotonic.

In previous maternal genetic studies, Pg synthesis function was significantly inhibited in F1 female offspring after Cd exposure during pregnancy, and Pg levels were still significantly inhibited in F2 females produced by mating the F1 female with healthy males, suggesting that abnormal steroid synthesis in offspring induced by Cd exposure during pregnancy can be transmitted to F2 offspring through maternal inheritance [8]. In addition, direct paternal exposure to Cd has also been shown to cause intergenerational effects of impaired hormone synthesis in ovarian GCs and transgenerational effects of reparative changes in hormone synthesis in ovarian GCs in offspring [26], which are also inconsistent with changes in this model. The above information suggests that different modes of exposure ( placental Cd exposure during pregnancy or oral Cd exposure) or different modes of inheritance (paternal or maternal inheritance) bring about different intergenerational and transgenerational effects that impact hormonal synthesis effects in offspring ovarian GCs. We believe that one of the main reasons for these differences is that the damage effects of parents caused by different exposure modes or different genetic patterns are different, thus showing different genetic effects.

### Paternal genetic effects of abnormal hormone synthesis and expression of hormone synthesis-related genes in ovarian GCs

Star, Cyp11a1, Cyp17a1 and Cyp19a1 are key genes in the hormone synthesis pathway and are often important targets of EDCs to that interfere with hormone synthesis. The protein expression levels of Star and Cyp11a1 were significantly decreased by acute bisphenol S exposure in mouse antral follicles in vitro [27]. The mRNA expression levels of Star and Cyp17a1 decreased in the ovaries of neonatal mice exposed to phthalate diester [28]. The protein expression of Cyp19a1 was significantly reduced in human GCs HGrC1 after 2 weeks of exposure to a mixture composed of bisphenol A, PCBs, benzo [a] pyrene, and PFOS [29]. Previous studies have also observed significant decreases in Star and Cyp11a1 during hormone synthesis impairment in ovarian GCs of offspring induced by Cd exposure. Therefore, we next investigated what changes occurred in hormone synthesis-related genes during paternal genetic effects of hormone synthesis disorders in offspring induced by Cd exposure during pregnancy.

In the F2 generation, the changes in StAR and CYP11A1 were similar to the changes in serum E_2_ levels, and they were speculated to be associated with E_2_ changes. Meanwhile, CYP17A1 was upregulated only in the high-dose group, which is not consistent with the pattern of hormonal alterations, and therefore it is presumed that it does not play a major role. In addition, the increase in Sf-1 protein expression caused upregulation of the mRNA expression of the other four factors, but the decrease in the mRNA levels of the four factors was found, thus, we speculated that Sf-1 may not play a major role in hormone synthesis disorders. We noticed that the mRNA and protein expression levels of some hormone synthesis-related genes are not consistent (e.g., Star, Cyp11a1, etc.). Complex regulatory mechanisms are known to exist during mRNA transcription and protein translation [30, 31], and therefore, protein translation regulatory mechanisms may be activated in the F2 generation, allowing protein levels to rise. This is consistent with our previous findings in maternally inheritance models [8].

In the F3 generation, we found that StAR and CYP11A1 also showed the same expression pattern, i.e., protein expression levels decreased in the 0.5 mg/kg group and increased in the 2.0 mg/kg group, suggesting self-repair in the 0.5 mg/kg group, but not in the 2.0 mg/kg group. However, the changes in Star and CYP11A1 protein levels were not consistent with the changes in serum hormone levels, indicating that StAR and CYP11A1 do not play a major role in the regulation of hormone levels. In addition, CYP17A1 protein levels were also highly expressed in the F3 generation high-dose group, indicating that CYP17A1 can be stably inherited in this model. The mRNA levels of Sf-1 increased in the 0.5 and 2.0 mg/kg groups, whereas the protein levels did not significantly change. These results suggest that the mRNA expression of Sf-1 in the F3 generation showed a reparative rise and had returned to normal by the protein levels. However, CYP17A1 and SF-1 are also inconsistent with the pattern of hormonal changes, so it is speculated that they also do not play a major role.

In summary, the changes of StAR and CYP11A1 may be important during the intergenerational effect of hormone synthesis disorders in ovarian GCs of offspring induced by Cd exposure during pregnancy; while in F3 generation, we have not found hormone synthesis-related genes that may be important.

### Paternal genetic effects of hormone synthesis disorders and expression of hormone synthesis-related miRNAs in ovarian GCs

MiRNA is a single-stranded RNA with 18–24 nucleotides that reduces the translation efficiency of a target mRNA by targeting complementary sites in the 3’ untranslated region (3’ UTR) of the mRNA [32]. In recent years, increasing evidence has shown that Cd exposure can cause changes in the expression profile of miRNAs, and differentially expressed miRNAs play a key role in regulating Cd damage to the kidney, liver, spleen, and reproductive system of animals [23–36]. In this study, 24 hormone synthesis-related miRNAs were selected by combining miRNA microarray, database prediction, relevant literature and previous findings. qRT‒PCR results suggested that the expression of 14 miRNAs were upregulated and 9 were downregulated in the F2 generation; 16 were upregulated and 6 were downregulated in the F3 generation. These results suggest that hormone synthesis-related miRNA expression is altered during paternal inheritance of hormone synthesis disorders in ovarian GCs of offspring induced by Cd exposure during pregnancy.

In the F2 generation, the expression of miR-146a-3p, miR-146a-5p, miR-146b-5p, miR-1839-5p, miR-27a-3p, miR-27b-3p, and miR-32-3p were upregulated, corresponding to changes in their target gene Star. Previous studies by our group have found that miR-27a-3p and miR-10b-5p can target Star to influence the regulation of E_2_ synthesis in the F1 generation following exposed to Cd during pregnancy [8], the results showed that miR-27a-3p was still important in the paternal genetic intergenerational effect of hormone synthesis disorders in ovarian GCs of offspring induced by Cd exposure during pregnancy, but miR-10b-5p was not. In addition, miR-27a-3p and miR-27b-3p can also target CYP11A1, which is also closely related to the downregulation of its expression. In addition, Li et al. showed that miR-146b can directly target the 3′ UTR of Cyp19a1 to prevent its translation [37]. MiR-146b-5p showed corresponding changes with Cyp19a1 in the F2 generation, suggesting that miR-146b-5p may be associated with alterations in Cyp19a1.

In the F3 generation, we also found that miR-146a-3p, miR-146a-5p, and miR-146b-5p may be involved in the regulation of Star, indicating that the above miRNAs may be important in both paternal intergenerational and transgenerational effects of hormone synthesis disorders in ovarian GCs induced by Cd exposure during pregnancy. In addition. miR-10b-5p, which did not play a major role in the F2 generation, may be associated with Star alterations in the F3 generation. The above results suggest that (1) miRNAs that play a major role between generations and across generations are different; (2) miRNAs that may play an important role are also different with different genetic patterns (paternal and maternal genetic models of Cd exposure during pregnancy); and (3) miRNAs that may be important are also different under different exposure modes, because under genetic models of previous paternal Cd exposure [26], we also found that certain miRNA expression patterns that may be different from those in this study model.

In summary, changes in the miR-27a-3p, miR-27b-3p, and miR-146 families may be important in paternal genetic intergenerational effects of Cd exposure-induced hormone synthesis disorders in ovarian GCs; changes in the miR-10b-5p and miR-146 families may be important in transgenerational effects of Cd-exposure-induced hormone synthesis disorders in ovarian GCs.

### Paternal genetic effects of hormone synthesis disorders and altered DNA methylation in ovarian GCs

DNA methylation is closely associated with the expression of hormone synthesis genes in ovarian GCs [38–43]. In this study, genome-wide DNA methylation sequencing was performed in GCs of the F2 control group and the 8.0 mg/kg group, and a total of 593 DMRs were found, but no differential methylation of hormone synthesis-related gene promoter regions was found. These results suggest that Cd exposure may affect the DNA methylation pattern of GCs in the F2 generation, but the DNA methylation changes may not be related to expression changes in hormone synthesis-related genes (Star, Cyp11a1, Cyp17a1, Cyp19a1, Sf-1). Notably, we found that the Adcy7 is hypomethylated, and Adcy7 is a type of adenylyl cyclase that regulates Star and Cyp11a1 expression through the cAMP/PKA signalling pathway. Further testing revealed that the mRNA expression levels of Adcy7 were elevated in the 8.0 mg/kg group compared to the control group in the F2 generation and in each dose group in the F3 generation. These results suggest that DNA methylation may be important in the development of paternal genetic intergenerational and transgenerational effects of Cd exposure-induced hormone synthesis disorders in ovarian GCs during pregnancy by altering the expression of Adcy7.

In addition, there are some questions that deserve our further consideration:

Alterations in miRNAs and DNA methylation may be important in E_2_ and Pg changes in the F2 and F3 generations. Indeed, miRNAs identified in this study that could play important roles are also extensively affected by DNA methylation. For example, aberrant DNA methylation of miR-146b-5p has been reported to be associated with the development of certain tumors [44]. In addition, aberrant DNA methylation in imprinting control regions can also affect the expression of miRNAs associated with imprinting control regions [45]. Moreover, changes in transcription factors and m^6^A modifications may also play an important role in the regulation of miRNA expression [20, 46]. Therefore, in this study, numerous altered miRNAs in the F2 and F3 generations may also be regulated by multiple mechanisms as described above. This needs to be further explored in future studies.

Increasing evidence suggests that vitamin, nutraceutical supplementation may play an important role in women ‘s health [47, 48]. Indeed, it has long been shown that vitamins E, C and A and β-carotene are effective in reducing Cd concentrations in organs and tissues and reducing Cd-induced toxic effects, including reproductive toxicity [49]. In addition, Cd-induced gonadal injury in male rats has also been reported to be relieved by supplementation with nutrients such as vitamins and carotenoids [50–52]. However, there is still a lack of relevant studies on whether the paternal genetic effects of hormone synthesis disorders in ovarian GCs of offspring induced by Cd exposure during pregnancy can be improved by vitamin supplementation or nutraceuticals, which is a research direction worthy of further attention.

In this study, we first investigated and elaborated that there may be intergenerational and transgenerational effects in the impairment of hormone synthesis in ovarian GCs of female offspring caused by paternal inheritance of Cd exposure during pregnancy. Important changes in hormone synthesis-related genes, miRNAs, and DNA methylation during this genetic effect were also reported for the first time. Of course, there are some limitations in this study: although genes or miRNAs that may be important in the process of paternal genetic intergenerational and transgenerational effects of Cd exposure during pregnancy induced hormone synthesis disorders in ovarian GCs have been obtained by analyzing the experimental results, a clear causal relationship between these changes and genetic effects cannot be determined and needs to be further studied in the future.

## Conclusion

In summary, there are paternal genetic intergenerational and transgenerational effects in ovarian GCs E_2_ and Pg synthesis disorders induced by Cd exposure during pregnancy. During intergenerational effects, the changes in Star and Cyp11a1 and the miR-27a-3p, miR-27b-3p and miR-146 families may be important. During transgenerational effects, changes in the miR-10b-5p, miR-211-3p, and miR-146 families may be important.

## Electronic supplementary material

Below is the link to the electronic supplementary material.

Supplementary Material 1: Table S1 Primer sequences.

## Data Availability

The relevant data and Additional file 1 is availed.

## References

[1] Järup L, Akesson A (2009). Current status of cadmium as an environmental health problem. Toxicol Appl Pharmacol.

[2] Omeje KO, Ezema BO, Okonkwo F, Onyishi NC, Ozioko J, Rasaq WA, Sardo G, Okpala COR (2021). Quantification of heavy metals and pesticide residues in widely consumed nigerian food crops using atomic absorption spectroscopy (AAS) and gas chromatography (GC). Toxins (Basel).

[3] Ding H, Li Z, Li X, Yang X, Zhao J, Guo J, Lu W, Liu H, Wang J (2022). FTO alleviates CdCl2-Induced apoptosis and oxidative stress via the AKT/Nrf2 pathway in bovine granulosa cells. Int J Mol Sci.

[4] Zhang W, Wu T, Zhang C, Luo L, Xie M, Huang H (2017). Cadmium exposure in newborn rats ovary induces developmental disorders of primordial follicles and the differential expression of SCF/c-kit gene. Toxicol Lett.

[5] Weng S, Wang W, Li Y, Li H, Lu X, Xiao S, Wu T, Xie M, Zhang W (2014). Continuous cadmium exposure from weaning to maturity induces downregulation of ovarian follicle development-related SCF/c-kit gene expression and the corresponding changes of DNA methylation/microRNA pattern. Toxicol Lett.

[6] Zhang W, Pang F, Huang Y, Yan P, Lin W (2008). Cadmium exerts toxic effects on ovarian steroid hormone release in rats. Toxicol Lett.

[7] Chandravanshi L, Shiv K, Kumar S (2021). Developmental toxicity of cadmium in infants and children: a review. Environ Anal Health Toxicol.

[8] Liu J, Zeng L, Zhuang S, Zhang C, Li Y, Zhu J, Zhang W (2020). Cadmium exposure during prenatal development causes progesterone disruptors in multiple generations via steroidogenic enzymes in rat ovarian granulosa cells. Ecotoxicol Environ Saf.

[9] Huang Y, Zhu J, Li H, Wang W, Li Y, Yang X, Zheng N, Liu Q, Zhang Q, Zhang W, Liu J (2020). Cadmium exposure during prenatal development causes testosterone disruption in multigeneration via SF–1 signaling in rats. Food Chem Toxicol.

[10] Parakh TN, Hernandez JA, Grammer JC, Weck J, Hunzicker-Dunn M, Zeleznik AJ, Nilson JH (2006). Follicle-stimulating hormone/cAMP regulation of aromatase gene expression requires beta-catenin. Proc Natl Acad Sci U S A.

[11] Chiminelli I, Spicer LJ, Maylem ERS, Caloni F (2022). In vitro effects of enniatin a on steroidogenesis and proliferation of bovine granulosa cells. Toxins (Basel).

[12] Yang R, Wang YM, Zhang LS, Zhang L, Zhao ZM, Zhao J, Peng SQ (2015). Delay of the onset of puberty in female rats by prepubertal exposure to T–2 toxin. Toxins (Basel).

[13] Skvortsova K, Iovino N, Bogdanović O (2018). Functions and mechanisms of epigenetic inheritance in animals. Nat Rev Mol Cell Biol.

[14] Hu Z, Shen WJ, Kraemer FB, Azhar S (2017). Regulation of adrenal and ovarian steroidogenesis by miR–132. J Mol Endocrinol.

[15] Gao S, Zhao J, Xu Q, Guo Y, Liu M, Zhang C, Schinckel AP, Zhou B (2022). MiR–31 targets HSD17B14 and FSHR, and miR–20b targets HSD17B14 to affect apoptosis and steroid hormone metabolism of porcine ovarian granulosa cells. Theriogenology.

[16] Zhu L, Jing J, Qin S, Zheng Q, Lu J, Zhu C, Liu Y, Fang F, Li Y, Ling Y (2021). miR–130a–3p regulates steroid hormone synthesis in goat ovarian granulosa cells by targeting the PMEPA1 gene. Theriogenology.

[17] Missaghian E, Kempná P, Dick B, Hirsch A, Alikhani-Koupaei R, Jégou B, Mullis PE, Frey BM, Flück CE (2009). Role of DNA methylation in the tissue-specific expression of the CYP17A1 gene for steroidogenesis in rodents. J Endocrinol.

[18] Monga R, Ghai S, Datta TK, Singh D (2011). Tissue-specific promoter methylation and histone modification regulate CYP19 gene expression during folliculogenesis and luteinization in buffalo ovary. Gen Comp Endocrinol.

[19] Sun Y, Lv Y, Li Y, Li J, Liu J, Luo L, Zhang C, Zhang W (2022). Maternal genetic effect on apoptosis of ovarian granulosa cells induced by cadmium. Food Chem Toxicol.

[20] Sun Y, Zong C, Liu J, Zeng L, Li Q, Liu Z, Li Y, Zhu J, Li L, Zhang C, Zhang W (2021). C-myc promotes miR–92a–2–5p transcription in rat ovarian granulosa cells after cadmium exposure. Toxicol Appl Pharmacol.

[21] Zhang W, Jia H (2007). Effect and mechanism of cadmium on the progesterone synthesis of ovaries. Toxicology.

[22] Sun Y, Wang W, Guo Y, Zheng B, Li H, Chen J, Zhang W (2019). High copper levels in follicular fluid affect follicle development in polycystic ovary syndrome patients: Population-based and in vitro studies. Toxicol Appl Pharmacol.

[23] Zoeller RT, Vandenberg LN (2015). Assessing dose-response relationships for endocrine disrupting chemicals (EDCs): a focus on non-monotonicity. Environ Health.

[24] Rattan S, Brehm E, Gao L, Niermann S, Flaws JA (2018). Prenatal exposure to di(2-ethylhexyl) phthalate disrupts ovarian function in a transgenerational manner in female mice. Biol Reprod.

[25] Nasiadek M, Danilewicz M, Klimczak M, Stragierowicz J, Kilanowicz A (2019). Subchronic exposure to cadmium causes persistent changes in the reproductive system in female wistar rats. Oxid Med Cell Longev.

[26] Sun Y, Zhang C, Luo L, Lin H, Liu C, Zhang W (2023). Paternal genetic intergenerational and transgenerational effects of cadmium exposure on hormone synthesis disorders in progeny ovarian granulosa cells. Environ Pollut.

[27] Amar S, Binet A, Téteau O, Desmarchais A, Papillier P, Lacroix MZ, Maillard V, Guérif F, Elis S (2020). Bisphenol S impaired human granulosa cell steroidogenesis in vitro. Int J Mol Sci.

[28] Lai FN, Liu JC, Li L, Ma JY, Liu XL, Liu YP, Zhang XF, Chen H, De Felici M, Dyce PW, Shen W (2017). Di (2-ethylhexyl) phthalate impairs steroidogenesis in ovarian follicular cells of prepuberal mice. Arch Toxicol.

[29] Samardzija Nenadov D, Tesic B, Fa S, Pogrmic-Majkic K, Kokai D, Stanic B, Andric N (2022). Long-term in vitro exposure of human granulosa cells to the mixture of endocrine disrupting chemicals found in human follicular fluid disrupts steroidogenesis. Toxicol In Vitro.

[30] Ji Q, Qu G, Liu B, Bai Y, Wang G, Chen R, Zheng X, Zhang Z, Yang Y, Wu C (2022). Evaluation of porcine GM-CSF during PRRSV infection in vitro and in vivo indicating a protective role of GM-CSF related with M1 biased activation in alveolar macrophage during PRRSV infection. Front Immunol.

[31] Wu Y, Qiu J, Chen S, Chen X, Zhang J, Zhuang J, Liu S, Yang M, Zhou P, Chen H, Ge J, Yu K, Zhuang J (2020). Crx is posttranscriptionally regulated by light stimulation in postnatal rat retina. Front Cell Dev Biol.

[32] Xu Z, Liu Q, Liu X, Yang M, Su Y, Wang T, Li D, Li F (2022). Integrated transcriptome analysis reveals mRNA-miRNA pathway crosstalk in roman laying hens’ immune organs induced by AFB1. Toxins (Basel).

[33] Fay MJ, Alt LAC, Ryba D, Salamah R, Peach R, Papaeliou A, Zawadzka S, Weiss A, Patel N, Rahman A, Stubbs-Russell Z, Lamar PC, Edwards JR, Prozialeck WC (2018). Cadmium nephrotoxicity is associated with altered microRNA expression in the rat renal cortex. Toxics.

[34] Fabbri M, Urani C, Sacco MG, Procaccianti C, Gribaldo L (2012). Whole genome analysis and microRNAs regulation in HepG2 cells exposed to cadmium. Altex.

[35] Chen M, Li X, Fan R, Yang J, Jin X, Hamid S, Xu S (2018). Cadmium induces BNIP3-dependent autophagy in chicken spleen by modulating miR–33-AMPK axis. Chemosphere.

[36] Wang W, Chen J, Luo L, Li Y, Liu J, Zhang W (2018). Effect of cadmium on kitl pre-mRNA alternative splicing in murine ovarian granulosa cells and its associated regulation by miRNAs. J Appl Toxicol.

[37] Li Q, Du X, Liu L, Liu H, Pan Z, Li Q. Upregulation of miR–146b promotes porcine ovarian granulosa cell apoptosis by attenuating CYP19A1. Domest Anim Endocrinol. 2021:106509. 10.1016/j.domaniend.2020.106509 32653739

[38] Yiqin C, Yan S, Peiwen W, Yiwei G, Qi W, Qian X, Panglin W, Sunjie Y, Wenxiang W (2022). Copper exposure disrupts ovarian steroidogenesis in human ovarian granulosa cells via the FSHR/CYP19A1 pathway and alters methylation patterns on the SF–1 gene promoter. Toxicol Lett.

[39] Li M, Zhou S, Wu Y, Li Y, Yan W, Guo Q, Xi Y, Chen Y, Li Y, Wu M, Zhang J, Wei J, Wang S (2021). Prenatal exposure to propylparaben at human-relevant doses accelerates ovarian aging in adult mice. Environ Pollut.

[40] Li H, Liu J, Sun Y, Wang W, Weng S, Xiao S, Huang H, Zhang W (2014). N-hexane inhalation during pregnancy alters DNA promoter methylation in the ovarian granulosa cells of rat offspring. J Appl Toxicol.

[41] Stocco C (2008). Aromatase expression in the ovary: hormonal and molecular regulation. Steroids.

[42] Lee L, Asada H, Kizuka F, Tamura I, Maekawa R, Taketani T, Sato S, Yamagata Y, Tamura H, Sugino N (2013). Changes in histone modification and DNA methylation of the StAR and Cyp19a1 promoter regions in granulosa cells undergoing luteinization during ovulation in rats. Endocrinology.

[43] Hosseini E, Mehraein F, Shahhoseini M, Karimian L, Nikmard F, Ashrafi M, Afsharian P, Aflatoonian R (2016). Epigenetic alterations of CYP19A1 gene in Cumulus cells and its relevance to infertility in endometriosis. J Assist Reprod Genet.

[44] Wolter M, Werner T, Malzkorn B, Reifenberger G (2016). Role of microRNAs located on chromosome arm 10q in malignant gliomas. Brain Pathol.

[45] Shin JY, Gupta MK, Jung YH, Uhm SJ, Lee HT (2011). Differential genomic imprinting and expression of imprinted microRNAs in testes-derived male germ-line stem cells in mouse. PLoS ONE.

[46] Li R, Zhu H, Li Q, Tang J, Jin Y, Cui H (2023). METTL3-mediated m^6^A modification of has_circ_0007905 promotes age-related cataract progression through miR–6749–3p/EIF4EBP1. PeerJ.

[47] Paul C, Laganà AS, Maniglio P, Triolo O, Brady DM (2016). Inositol’s and other nutraceuticals’ synergistic actions counteract insulin resistance in polycystic ovarian syndrome and metabolic syndrome: state-of-the-art and future perspectives. Gynecol Endocrinol.

[48] Chiaffarino F, Parazzini F, Surace M, Benzi G, Chiantera V, La Vecchia C (2003). Diet and risk of seromucinous benign ovarian cysts. Eur J Obstet Gynecol Reprod Biol.

[49] Sitek A, Kozłowska L (2022). The role of well-known antioxidant vitamins in the prevention of cadmium-induced toxicity. Int J Occup Med Environ Health.

[50] Abdel-Wahab A, Hassanin KMA, Mahmoud AA, Abdel-Badeea WIE, Abdel-Razik AH, Attia EZ, Abdelmohsen UR, Abdel Aziz RL, Najda A, Alanazi IS, Alsharif KF, Abdel-Daim MM, Mahmoud MO (2021). Physiological roles of red carrot methanolic extract and vitamin E to abrogate cadmium-induced oxidative challenge and apoptosis in rat testes: involvement of the Bax/Bcl–2 ratio. Antioxid (Basel).

[51] Chen Z, Zuo Z, Chen K, Yang Z, Wang F, Fang J, Cui H, Guo H, Ouyang P, Chen Z, Huang C, Geng Y, Liu W, Deng H (2022). Activated Nrf–2 pathway by vitamin E to attenuate testicular injuries of rats with sub-chronic cadmium exposure. Biol Trace Elem Res.

[52] Liu XR, Wang YY, Fan HR, Wu CJ, Kumar A, Yang LG (2016). Preventive effects of β-cryptoxanthin against cadmium-induced oxidative stress in the rat testis. Asian J Androl.

