# Supplementary material for: Paternal genetic effects of cadmium exposure during pregnancy on hormone synthesis disorders in ovarian granulosa cells of offspring
Source: J Ovarian Res. 2023 May 16;16:98. doi: 10.1186/s13048-023-01175-5 (PMC10186638; doi:10.1186/s13048-023-01175-5)
Supplement: Supplementary file 1 — Supplementary Material 1: Table S1 Primer sequences. [file 13048_2023_1175_MOESM1_ESM.docx]

Table S1 Primer Sequences

| Gene name | Primer sequences |
| --- | --- |
| *β-actin* | Forward:5’-CGTTGACATCCGTAAAGAC-3’ |
|  | Reverse:5’-TAGGAGCCAGGGCAGTA-3’ |
| *Star* | Forward:5’-CAAACTCACGTGGCTGCTCAGTA-3’ |
|  | Reverse:5’-GCAAGTGGCTGGCGAACTCTA-3’ |
| *Sf-1* | Forward:5’-CTCAGAGACACGAAGCATTACCAAC-3’ |
|  | Reverse:5’-ATCCATCCGTGCTTTATCCTGA-3’ |
| *Cyp11a1* | Forward:5’-GGGCATTTGAGGGTGGTGT-3’ |
|  | Reverse:5’-AGGGAGACAGGATGAAAGAGAGG-3’ |
| *Cyp19a1* | Forward:5’-CACTCAAGGGCAAGATGATAAGG-3’ |
|  | Reverse:5’-TGTAAGGGTGCTGGAATGGA-3’ |
| U6 | Forward:5’-GGAACGATACAGAGAAGATTAGC-3’ |
|  | Reverse:5’-TGGAACGCTTCACGAATTTGCG-3’ |
| 1. rno-miR-10b-5p | 1. 5’ CCCTGTAGAACCGAATTTGTGT 3’ |
| 1. rno-miR-125b-1-3p | 1. 5’ ACGGGTTAGGCTCTTGGGAGCT 3’ |
| 1. rno-miR-138-5p | 1. 5’ AGCTGGTGTTGTGAATCAGGCCG 3’ |
| 1. rno-miR-146a-3p | 1. 5’ ACCTGTGAAGTTCAGTTCTTT 3’ |
| rno-miR-146a-5p | 1. 5’ TGAGAACTGAATTCCATGGGTT 3’ |
| 1. rno-miR-146b-5p | 1. 5’ TGAGAACTGAATTCCATAGGCTGT 3’ |
| 1. rno-miR-152-3p | 1. 5’ TCAGTGCATGACAGAACTTGG 3’ |
| 1. rno-miR-1839-5p | 1. 5’ AAGGTAGATAGAACAGGTCTTG 3’ |
| 1. rno-miR-185-5p | 1. 5’ TGGAGAGAAAGGCAGTTCCTGA 3’ |
| 1. rno-miR-1896 | 1. 5’ TGGTGGGTGAGGAGGAGG 3’ |
| 1. rno-miR-207 | 1. 5’ AGTACTTCTCCTGGCTCTCCTCC 3’ |
| 1. rno-miR-210-3p | 1. 5’ AAGGTTGTCTGTGCGTGTGAC 3’ |
| 1. rno-miR-211-3p | 1. 5’ AAGCACGGCAAGGACAGCA 3’ |
| 1. rno-miR-212-5p | 1. 5' AACCACTACCTTGGCTCTAGACT 3’ |
| 1. rno-miR-24-3p | 1. 5’AATTGTACTGGCTCAGTTCAGCA 3’ |
| 1. rno-miR-27a-3p | 1. 5’ TTCACAGTGGCTAAGTTCCGC 3’ |
| 1. rno-miR-27a-5p | 1. 5’ AAGCACAGGGCTTAGCTGCT 3’ |
| 1. rno-miR-27b-3p | 1. 5’ AGCGCCTTTCACAGTGGCTA 3’ |
| 1. rno-miR-32-3p | 1. 5’ AAGAGCGTGCAATTTAGTGTGTG 3’ |
| 1. rno-miR-3558-5p | 1. 5’ AACAGTGCCATAGAAGTCATCCC 3’ |
| 1. rno-miR-465-5p | 1. 5’ CGCTATTTAGAACGGTGCTGGTGTG 3’ |
| 1. rno-miR-486 | 1. 5’ CCTAGATCCTGTACTGAGCTGCC 3’ |
| 1. rno-miR-673-5p | 1. 5’ ACCTCATACTCACAGCTCCGG 3’ |
| 1. rno-miR-702-3p | 1. 5’ TGCCCACCCTTTACCCCACTCCA 3’ |
